# Osteosarcopenia as a Predictor of Histopathologic Response to Neoadjuvant Chemoradiotherapy in Esophageal Cancer: a Retrospective Cohort Study

**DOI:** 10.1007/s00423-025-03687-8

**Published:** 2025-03-25

**Authors:** Yuki Hirase, Ken Sasaki, Yusuke Tsuruda, Masataka Shimonosono, Yasuto Uchikado, Daisuke Matsushita, Takaaki Arigami, Nobuhiro Tada, Kenji Baba, Yota Kawasaki, Takao Ohtsuka

**Affiliations:** Department of Digestive Surgery, Kagoshima University Graduate School of Medical and Dental Sciences, 8-35-1 Sakuragaoka, Kagoshima-shi, 890-8520 Japan

**Keywords:** Esophageal cancer, Osteosarcopenia, Prognosis

## Abstract

**Purpose:**

Predicting chemoradiotherapy (CRT) response in esophageal cancer is essential as outcomes vary among patients. This study aimed to evaluate the impact of osteosarcopenia on the effectiveness of neoadjuvant CRT (NACRT).

**Methods:**

Ninety-five patients with advanced esophageal cancer who underwent surgical resection post-NACRT were included. Sarcopenia and osteopenia were determined using pre-NACRT skeletal muscle index and bone density at L3 and Th11 levels. Patients were categorized based on osteosarcopenia status.

**Results:**

Thirty-seven patients (39%) had osteosarcopenia. Among tumors, 49 (51.6%) were grade 1 (non-responders), 12 (12.6%) were grade 2, and 34 (35.8%) were grade 3 (responders). NACRT was significantly more effective in patients with above-median body mass index, shallow tumor depth, low squamous cell carcinoma antigen levels, and without osteosarcopenia. Osteosarcopenia was independently correlated with the histopathologic response to NACRT. No significant differences in overall or relapse-free survival were observed based on osteosarcopenia status.

**Conclusion:**

Osteosarcopenia may predict NACRT response in esophageal cancer.

## Introduction

Esophageal cancer is the seventh most common cancer worldwide and the sixth most common cause of cancer-related mortality [1]. Currently, the standard treatment for locally advanced esophageal cancer in Western countries is neoadjuvant chemoradiation therapy (NACRT), followed by surgery [2]. However, chemoradiation therapy (CRT) for esophageal cancer is associated with severe side effects, and surgery is highly invasive. The prognosis of esophageal cancer remains unsatisfactory due to a high postoperative recurrence rate [3]. CRT is a widely applied treatment for early-stage and locally advanced esophageal cancer, as well as for palliative treatment [4]. Notably, patients with esophageal cancer who respond to CRT exhibit improved survival rates compared to non-responders, underscoring the importance of predicting and selecting responders in advance [5, 6]. However, no reliable tool has been identified to predict response to CRT in esophageal cancer.

Sarcopenia and osteopenia have garnered increasing attention in recent years [7, 8]. Sarcopenia is characterized by decreased skeletal muscle mass and strength and is associated with poor prognosis in patients with esophageal cancer [9, 10]. Osteopenia is characterized by low bone mineral density, and several studies have demonstrated an association between osteopenia and prognosis in various malignancies, including esophageal cancer [11, 12]. “Osteosarcopenia,” a condition involving sarcopenia and osteopenia, has been recognized as a prognostic factor in several patients with cancer [7, 13]. However, few studies have been conducted regarding CRT and osteosarcopenia in patients with esophageal cancer, and the clinical impact of osteosarcopenia on CRT remains unclear.

Therefore, the objectives of this study were to investigate sarcopenia, osteopenia, and osteosarcopenia in patients with advanced esophageal cancer who underwent curative resection after NACRT by examining their correlation with the histopathological treatment response, and to evaluate the clinical significance of osteosarcopenia as a new predictive marker of treatment response.

## Material and methods

### Participants

The study retrospectively analyzed 95 patients (men: 85, women: 10; age range: 43–77 [median: 64] years) with advanced esophageal cancer who underwent NACRT followed by surgical resection at Kagoshima University Hospital between April 2007 and December 2020. Patients were classified and staged according to the TNM classification for esophageal cancer [14]. The median follow-up period was 41 months.

Informed consent was obtained from patients using an “opt-out” method. This retrospective study was approved by the Kagoshima University Ethics Committee (approval no. 240061; approval date: July 25, 2024).

### Radiology

Patients were treated with 6 MV or 10 MV external photon radiotherapy, with total doses ranging from 40 to 41.4 Gy. The clinical target volumes included primary tumors, metastatic lymph nodes (LNs), and regional LNs. Prophylactic irradiation was administered to noninvasive regional LNs including the bilateral supraclavicular and peritumoral LNs.

### Chemotherapy

All patients received either with cisplatin and 5-fluorouracil plus radiation therapy (CF-RT) or docetaxel, cisplatin, and 5-fluorouracil plus radiation therapy (DCF-RT). Therapy comprised two courses of CF (cisplatin, 70 mg/m^2^/day, day 1; 5-FU, 700 mg/m^2^/day, days 1–5) repeated every 4 weeks or low-dose CF (cisplatin, 4 mg/m^2^/day 1-h infusion, days 1–5, 8–12, 15–19, 22–26; 5-FU, 200 mg/m^2^/day continuous infusion, days 1–5, 8–12, 15–19, 22–26); in the DCF-RT group, chemotherapy regimen consisted of DCF (docetaxel, 30 mg/m^2^/day, day 1; cisplatin, 7 mg/m^2^/day, days 1–5 and 8–12; 5-FU, 350 mg/m^2^/day, days 1–5 and 8–12) repeated every 2 weeks.

### Surgery

Open transthoracic, thoracoscopic, or mediastinal esophagectomy was scheduled 8–10 weeks after completion of NACRT. The proportion of thoracoscopic procedures performed increased annually according to the surgeon's proficiency. Indications for mediastinoscopic esophagectomy included advanced age, a history of lobectomy, or severe chest adhesions due to pleurisy or inflammatory disease. Histopathological evaluation was conducted based on the Japanese Classification of Esophageal Cancer by the Japan Esophageal Society. HE-stained specimens from resected tissues were classified as follows: Grade 3 (no viable cancer cells detected), Grade 2 (viable cancer cells occupy less than one-third of the entire tissue), and Grade 1 (viable cancer cells occupy one-third or more of the entire tissue).

### Follow-up

Blood tests and computed tomography (CT) scans were performed every 3 months esophagogastroscopy was conducted annually.

### Definition of sarcopenia, osteopenia, and osteosarcopenia

CT images obtained before NACRT were analyzed using the Volume Analyzer SYNAPSE VINCENT imaging analysis system (Fujifilm Medical, Tokyo, Japan) to evaluate body composition indices.

Sarcopenia was defined as a decrease in the skeletal muscle index (SMI) measured at the L3 vertebral level before NACRT. The tissue Hounsfield unit threshold for skeletal muscle was set at a range of −29 to 150 HU [15] . SMI (cm^2^/m^2^) was calculated by dividing the skeletal muscle area (cm^2^) by the square of the height (m^2^) [13] . Cutoff values for SMI were 52 for males and 38 for females [16].

Osteopenia was defined as the actual BMD being lower than the calculated standard BMD (males: 308.82 - 2.49 × age [years]; females: 311.84 - 2.41 × age [years]) [13]. BMD was assessed before NACRT by examining the average pixel density within an elliptical core at the Th11 vertebral level.

Osteosarcopenia was defined as the coexistence of sarcopenia and osteopenia.

### Blood markers for nutrition and systemic inflammatory response

In this study, the prognostic nutrition index (PNI), neutrophil–lymphocyte ratio (NLR), platelet–lymphocyte ratio (PLR), lymphocyte–monocyte ratio (LMR), C-reactive protein (CRP)/albumin ratio (CAR), pan-immune-inflammation value (PIV), and systemic immune-inflammation index (SII) were utilized as indicators of nutritional and systemic inflammatory responses. Blood samples were collected before NACRT. Neutrophils, lymphocytes, monocytes, and platelets were counted using an XN-20 automated hematology analyzer (Sysmex Corporation, Kobe, Japan), and CRP and albumin levels were measured using a JCA-ZS050 automated analyzer (JEOL, Tokyo, Japan).

PNI was calculated by adding the serum albumin value multiplied by 10 and the lymphocyte count multiplied by 0.005 [17]; NLR was calculated by dividing the neutrophil count by the lymphocyte count [17]; PLR was calculated by dividing the platelet count by the lymphocyte count [17]; LMR was calculated by dividing the lymphocyte count by the monocyte count [17]; CAR was calculated by dividing the CRP by albumin [17]; PIV was calculated by multiplying the neutrophil, monocyte, and platelet counts by the lymphocyte count [18]; and SII was calculated by multiplying the neutrophil and platelet counts by the lymphocyte count [17].

### Statistical analyses

The cutoff values for age, body mass index (BMI), PNI, NLR, PLR, LMR, CAR, PIV, and SII were based on median values. The relationships between the histopathological treatment effect and clinical factors were evaluated using the chi-square test, Fisher's exact test, and Wilcoxon rank-sum test. Overall survival (OS) was defined as the time from surgery to death or final follow-up. Relapse-free survival (RFS) was defined as the time from surgery to the date of confirmed recurrence or secondary malignancy, death from any cause, or last follow-up. Kaplan-Meier survival curves were generated and prognostic differences were determined using log-rank tests. The factors were identified using univariate and multivariate analysis (logistic regression modeling). All data were analyzed using JMP software (SAS Institute Inc., Heidelberg, Germany). Statistical significance was set at p<0.05.

## Results

### Clinicopathological characteristics of patients

The clinicopathological characteristics of the 95 participants are summarized in Table 1. The median age was 64 (range: 43–77) years. The tumors were located in the upper, middle, and lower thoracic regions in 27 (28%), 46 (48%), and 22 (23%) patients, respectively. Regarding the depth of tumor invasion, nine (9%), seven (7%), 76 (80%), and three (3%) patients were clinically classified as T1, T2, T3, and T4, respectively. For lymph node metastasis, nine (9%), 31 (31%), 36 (38%), and 19 (20%) patients were clinically classified as N1, N2, N3, and N4, respectively. Regarding distant metastasis to the supraclavicular lymph nodes, 68 (72%) and 27 (28%) patients had M0 and M1 disease, respectively. Thirty-eight (40%) and 57 (60%) patients underwent CF-RT and DCF-RT, respectively. Histopathologic efficacy determinations were Grade 1, Grade 2, and Grade 3 in 49 (52%), 12 (13%), and 34 (36%) patients, respectively, with median pre-NACRT PNI, NLR, PLR, LMR, CAR, PIV, SII, p53, and squamous cell carcinoma (SCC) antigen values of 49.1 (range: 0–62 .6), 2.05 (range: 0.78–5.73), 142.5 (range: 20.1–464.6), 4.49 (range: 1.59–14.88 g), 0.03 (range: 0–2.20), 168.5 (range: 38.0–2586.0), 488622 (range: 115986–2547851), 0 .4 (range: 0.4–264.0), and 1.4 (range: 0.4–6.0), respectively.

**Table 1:** Clinicopathological characteristics of patients (*n* = 95)

Characteristic	n
Sex, male/female	85/10
Median age (range), years	64 (43–77)
Median BMI (range), kg/m^2^	21.6(14.3-–30.5)
Tobacco use, Yes/No	64/31
Alcohol use, Yes/No	78/17
Tumor location, Ut / Mt / Lt	27/46/22
Depth of tumor invasion, cT^a^1/cT2/cT3/cT4	9/7/76/3
Lymph node metastasis, cN^a^0/cN1/cN2/cN3	9/31/36/19
Distant lymph node metastasis^b^, cM^a^0/cM1	68/27
cStage^a^, I/II/III/IV	5/9/42/39
Preoperative treatment, CF-RT/DCF-RT	38/57
pGrade, 1/2/3	49/12/34
Median PNI (range)	49.1 (0–62.6)
Median NLR (range)	2.05 (0.78–5.73)
Median PLR (range)	142.5 (20.1–464.6)
Median LMR (range)	4.49 (1.59–14.88)
Median CAR (range)	0.03 (0.00–2.20)
Median PIV (range)	168.5 (38.0–2586.0)
Median SII (range)	488622 (115986–2547851)
Median p53 (range)	0.4 (0.4–264)
Median SCC antigen (range)	1.4 (0.4–6.0)

*BMI* body mass index; *CAR* C-reactive protein/albumin ratio; *CF-RT* cisplatin+5FU+radiotherapy; *DCF-RT* docetaxel+cisplatin+5FU+radiotherapy; *LMR* lymphocyte–monocyte ratio; *Lt* lower thoracic esophagus; *Mt* middle thoracic esophagus; *NLR* neutrophil–lymphocyte ratio; *PIV* pan-immune-inflammation value; *PLR* platelet–lymphocyte ratio; *PNI* prognostic nutritional index; *SCC* squamous cell carcinoma; *SII* systemic immune-inflammation index; *Ut* upper thoracic esophagus

^a^UICC 8^th^ edition

^b^supraclavicular lymph node metastasis

### Assessment of sarcopenia, osteopenia, and osteosarcopenia

**Fig. 1:**
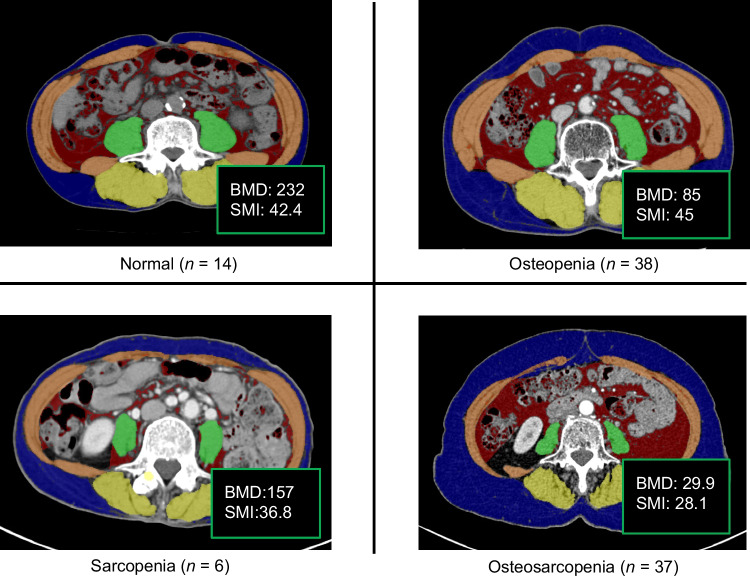
Representative computed tomography images in a normal patient, and in patients with sarcopenia only, osteopenia only, and osteosarcopenia

Fig. 1 presents representative CT images of patients classified as normal, sarcopenia only, osteopenia only, and osteosarcopenia, which were identified in 14 (15%), six (6%), 38 (40%), and 37 (39%) patients, respectively.

### Relationship between histopathologic response and clinical factors

Significant correlations were identified between histopathologic treatment response and BMI, tumor depth, SCC antigen, and osteosarcopenia (p < 0.01, p = 0.01, p < 0.01, and p < 0.01, respectively; Table 2). No correlations were identified between sex, age, smoking, alcohol consumption, tumor location, lymph node metastasis, distant metastasis, stage, preoperative treatment, PNI, NLR, PLR, LMR, CAR, PIV, SII, p53, and histopathological treatment response (all p > 0.05; Table 2).

**Table 2:** Relationship between histopathological response and clinical characteristics of patients (*n* = 95)

Characteristic	pGrade		*p*-value
	pGrade 1 (*n *= 49)	pGrade 2/3 (*n *= 46)	
Sex, male/female	45/4	40/6	0.60
Median age (range), years	64 (44–77)	64 (43–76)	0.42
Median BMI (range), kg/m^2^	20.7 (14.3–26.6)	22.4 (15.6–30.5)	<0.01
Tobacco use, Yes/No	35/14	29/17	0.57
Alcohol use, Yes/No	43/6	35/11	0.14
Tumor location, Ut / Mt / Lt	14/21/14	8/25/13	0.37
Depth of tumor invasion, cT^a^1/cT2/cT3/cT4	2/1/43/3	7/6/33/0	0.01
Lymph node metastasis, cN^a^0/cN1/cN2/cN3	5/14/20/10	4/17/16/9	0.84
Distant lymph node metastasis^b^, cM^a^0/cM1	36/13	32/14	0.67
cStage^a^, I/II/III/IV	1/4/23/21	4/5/19/18	0.47
Preoperative treatment, CF-RT/DCF-RT	20/29	18/28	1.00
Median PNI (range)	48.6 (0–62.6)	49.4 (0–58.5)	0.44
Median NLR (range)	1.99 (0.78–5.73)	2.08 (1.10–5.51)	0.92
Median PLR (range)	145.9 (20.1–274.6)	135.7 (56.4–464.6)	0.51
Median LMR (range)	4.48 (1.59–8.79)	4.49 (2.09–14.89)	0.54
Median CAR (range)	0.04 (0.00–0.65)	0.04 (0.00–2.21)	0.58
Median PIV (range)	185.0 (38.0–2586.1)	166.7 (79.0–1120.1)	0.37
Median SII (range)	482985 (115986–2547852)	518721 (208090–214537)	0.97
Median p53 (range)	0.40 (0.4–264)	0.40 (0.40–243)	0.68
Median SCC antigen (range)	1.65 (0.6–6.0)	1.25 (0.4–3.5)	<0.01
Osteosarcopenia, presence/absence	30/19	7/39	<0.01

*BMI* body mass index; *CAR* C-reactive protein/albumin ratio; *CF-RT* cisplatin+5FU+radiotherapy; *DCF-RT* docetaxel+cisplatin+5FU+radiotherapy; *LMR* lymphocyte–monocyte ratio; *Lt* lower thoracic esophagus; *Mt* middle thoracic esophagus; *NLR* neutrophil–lymphocyte ratio; *PIV* pan-immune-inflammation value; *PLR* platelet–lymphocyte ratio; *PNI* prognostic nutritional index; *SCC* squamous cell carcinoma; *SII* systemic immune-inflammation index; *Ut* upper thoracic esophagus

^a^UICC 8^th^ edition, ^b^supraclavicular lymph node metastasis

### Prognostic analysis determined by histopathological response

**Fig. 2:**
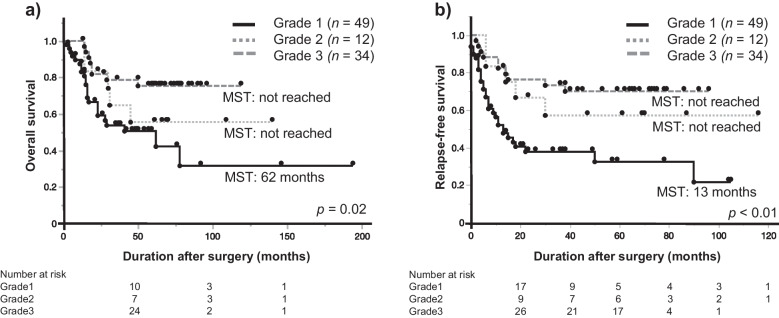
Kaplan–Meier survival curves after esophageal cancer surgery with histopathological effects. **(a)** Overall survival; (**b**) Relapse-free survival

The median OS of 95 patients was 41 months. The median OS was highest for grade 3 and significantly lower for grade 1 (median OS: not reached, 62 months) (p = 0.02; Fig. 2a). Similarly, the median RFS was the highest for grade 3 and significantly lower for grade 1 (median RFS: not reached, 13 months) (p < 0.01; Fig. 2b).

Univariate analysis of histopathologic response, nutritional inflammation markers, and clinical characteristics revealed that tumor depth, SCC antigen level, and osteosarcopenia were significantly correlated with the histopathologic response to treatment (p < 0.01, p = 0.01, and p < 0.01, respectively; Table 3). However, multivariate analysis identified osteosarcopenia as the only independent predictive factor correlated with the histopathologic treatment response (p < 0.01; Table 3).

**Table 3:** Univariate and multivariate analyses of factors associated with histopathological responses of patients (*n* = 95)

Independent factor	Univariate analysis			Multivariate analysis		
	Odds ratio	95% CI	*p*-value	Odds ratio	95% CI	*p*-value
Sex, Female	1.93	0.52–7.22	0.33			
Age, >64 years	1.29	0.55–3.02	0.55			
BMI, >21.6 kg/m^2^	2.03	0.86–4.79	0.10			
Tobacco use, Yes	1.01	0.42–2.50	0.97			
Alcohol use, No	1.32	0.45–3.86	0.61			
Tumor location, Lt	1.07	0.43–1.72	0.87			
Depth of tumor invasion, cT^a^1–2	5.36	1.67–17.1	<0.01	3.15	0.87–11.4	0.08
Lymph node metastasis, cN^a^1–3	1.13	0.26–4.83	0.87			
Distant lymph node metastasis^b^, cM^a^1	1.08	0.43–2.72	0.87			
Preoperative treatment, DCF-RT	1.36	0.57–3.24	0.49			
PNI, >49.1	1.40	0.60–3.25	0.44			
NLR, >2.05	1.66	0.70–3.91	0.25			
PLR, <142.5	2.01	0.85–4.78	0.11			
LMR, >4.49	1.06	0.45–2.49	0.89			
CAR, <0.03	1.93	0.82–4.56	0.13			
PIV, >168.5	1.40	0.60–3.25	0.44			
SII, <488622	1.37	0.58–3.22	0.47			
SCC, ≤1.5	3.35	1.31–8.58	0.01	2.46	0.87–7.00	0.09
p53, >0.40	1.51	0.60–3.78	0.38			
Osteosarcopenia, absence	6.39	2.19–18.7	<0.01	5.67	1.86–17.3	<0.01

*BMI* body mass index; *CAR* C-reactive protein/albumin ratio; *DCF-RT* docetaxel+cisplatin+5FU+radiotherapy; *LMR* lymphocyte–monocyte ratio; *Lt* lower thoracic esophagus; *NLR* neutrophil–lymphocyte ratio; *PIV* pan-immune-inflammation value; *PLR* platelet–lymphocyte ratio; *PNI* prognostic nutritional index; *SCC* squamous cell carcinoma; *SII* systemic immune-inflammation index

^a^UICC 8^th^ edition

^b^supraclavicular lymph node metastasis

### Prognostic analysis determined by osteosarcopenia

**Fig. 3:**
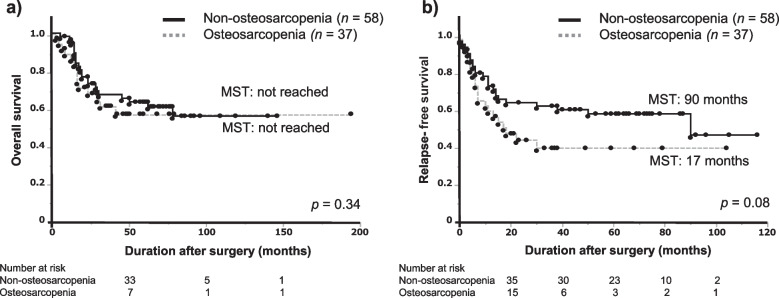
Kaplan–Meier survival curves after esophageal cancer surgery in the presence or absence of osteosarcopenia. **(a)** Overall survival; (**b**) Relapse-free survival

The median OS did not demonstrate a statistically significant difference between the osteosarcopenia and non-osteosarcopenia groups (p = 0.37; Fig. 3a). Although the median RFS was shorter in the osteosarcopenia group than in the non-osteosarcopenia group, no significant difference was observed (p = 0.08; Fig. 3b).

The univariate analysis of OS with nutritional inflammation markers and clinical characteristics revealed that distant metastasis, preoperative treatment, and pathological response rates were significantly associated with OS (p < 0.01, p = 0.02, and p < 0.01, respectively; Table 4). Multivariate analysis further identified distant metastasis, preoperative treatment, and pathological response rates as independent prognostic factors for OS (p = 0.02, p = 0.04, and p < 0.01, respectively; Table 4). The univariate analysis of RFS with nutritional inflammation markers and clinical characteristics showed significant correlations of RFS with tumor invasion depth, preoperative treatment, PLR, LMR, PIV, SCC, and pathological response rates (p = 0.01, p = 0.04, p = 0.01, p = 0.02, p = 0.04, p = 0.04, and p < 0.01, respectively; Table 5). Multivariate analysis identified tumor invasion depth, preoperative treatment, PLR, and pathological response rates as independent prognostic factors for RFS (p = 0.04, p = 0.02, p = 0.02, and p < 0.01, respectively; Table 5).

**Table 4.** Univariate and multivariate analysis of overall survival in patients with esophageal cancer (*n* = 95)

Independent factor	Univariate analysis			Multivariate analysis		
	Odds ratio	95% CI	*p*-value	Odds ratio	95% CI	*p*-value
Sex, female	1.34	0.62–2.90	0.46			
Age, >64 years	1.09	0.61–1.92	0.78			
BMI, <21.6 kg/m^2^	1.34	0.75–2.40	0.32			
Tobacco use, Yes	0.94	0.51–1.76	0.87			
Alcohol use, Yes	1.45	0.75–2.82	0.27			
Tumor location, Lt	0.80	0.43–1.50	0.49			
Depth of tumor invasion, cT^a^3–4	1.18	0.63–2.22	0.60			
Lymph node metastasis, cN^a^0	1.45	0.61–3.46	0.40			
Distant lymph node metastasis^b^, cM^a^1	3.07	1.48–6.37	<0.01	2.61	1.12–4.16	0.02
Preoperative treatment, DCF-RT	2.16	1.09–4.30	0.02	0.52	0.29–0.96	0.04
PNI, >49.1	1.56	0.87–2.78	0.13			
NLR, >2.05	1.79	0.98–3.28	0.06			
PLR, >142.5	1.68	0.92–3.06	0.09			
LMR, <4.49	1.79	0.99–3.27	0.06			
CAR, >0.03	1.38	0.75–2.53	0.31			
PIV, >168.5	1.51	0.83–2.74	0.18			
SII, >488622	1.38	0.76–2.51	0.28			
SCC, >1.5	1.61	0.89–2.90	0.11			
p53, >0.40	1.27	0.65–2.51	0.49			
Histopathological responses, pGrade1	2.61	1.44–4.96	<0.01	3.03	1.60–5.73	<0.01
Osteosarcopenia, presence	1.38	0.75–2.53	0.29			

*BMI* body mass index; *CAR* C-reactive protein/albumin ratio; *DCF-RT* docetaxel+cisplatin+5FU+radiotherapy; *LMR* lymphocyte–monocyte ratio; *Lt* lower thoracic esophagus; *NLR* neutrophil–lymphocyte ratio; *PIV* pan-immune-inflammation value; *PLR* platelet–lymphocyte ratio; *PNI* prognostic nutritional index; *SCC* squamous cell carcinoma; *SII* systemic immune-inflammation index

^a^UICC 8^th^ edition

^b^supraclavicular lymph node metastasis

**Table 5.** Univariate and multivariate analysis of relapse-free survival in patients with esophageal cancer (*n* = 95)

Independent factor	Univariate analysis			Multivariate analysis		
	Odds ratio	95% CI	*p*-value	Odds ratio	95% CI	*p*-value
Sex, male	1.01	0.40–2.55	0.99			
Age, <64 years	1.09	0.61–1.94	0.78			
BMI, <21.6 kg/m^2^	1.33	0.74–2.39	0.33			
Tobacco use, No	1.14	0.62–2.10	0.67			
Alcohol use, Yes	1.45	0.65–3.24	0.37			
Tumor location, Lt	0.92	0.49–1.76	0.81			
Depth of tumor invasion, cT^a^3–4	13.40	1.84–97.50	0.01	8.56	1.10–66.60	0.04
Lymph node metastasis, cN^a^1–3	1.25	0.45–3.47	0.67			
Distant lymph node metastasis^b^, cM^a^1	1.73	0.94–3.20	0.08			
Preoperative treatment, CF-RT	1.81	1.01–3.23	0.04	2.10	1.12–3.93	0.02
PNI, <49.1	1.27	0.71–2.27	0.42			
NLR, >2.05	1.33	0.74–2.39	0.34			
PLR, >142.5	2.18	1.19–4.01	0.01	2.29	1.17–4.48	0.02
LMR, <4.49	2.10	1.15–3.82	0.02	1.80	0.88–3.68	0.11
CAR, >0.03	1.59	0.86–2.94	0.14			
PIV, >168.5	1.82	1.00–3.31	0.04	1.01	0.51–2.00	0.98
SII, >488622	1.58	0.87–2.85	0.13			
SCC, >1.5	1.79	1.00–3.21	0.04	1.09	0.58–2.05	0.79
p53, >0.40	1.28	0.65–2.49	0.48			
Histopathological responses, pGrade1	2.92	1.56–5.44	<0.01	2.84	1.45–5.57	<0.01
Osteosarcopenia, presence	1.66	0.92–2.98	0.09			

*BMI* body mass index; *CAR* C-reactive protein/albumin ratio; *CF-RT* cisplatin+5FU+radiotherapy; *LMR* lymphocyte–monocyte ratio; *Lt* lower thoracic esophagus; *NLR* neutrophil–lymphocyte ratio; *PIV* pan-immune-inflammation value; *PLR* platelet–lymphocyte ratio; *PNI* prognostic nutritional index; *SCC* squamous cell carcinoma; *SII* systemic immune-inflammation index

^a^UICC 8^th^ edition

^b^supraclavicular lymph node metastasis

## Discussion

In recent years, research has focused on predicting the histopathologic response to NACRT in esophageal cancer [19, 20]. This study investigated body composition to evaluate the relationship between osteosarcopenia and the histopathologic response to NACRT in patients with esophageal cancer and found the following: (1) The incidence of osteosarcopenia was higher in patients with advanced esophageal cancer. (2) There was a significant correlation between histopathologic treatment efficacy and OS and RFS. (3) There was no significant correlation between presence or absence of osteosarcopenia and OS and RFS. (4) A significant correlation was observed between the histopathological treatment response and BMI, clinical tumor depth, SCC antigen, and osteosarcopenia. (5) Multivariate analysis revealed that osteosarcopenia was an independent factor associated with histopathological treatment response. To our knowledge, this is the first study to indicate the potential clinical utility of osteosarcopenia as a predictor of treatment response to NACRT in esophageal cancer.

Patients with advanced cancer frequently experience malabsorption and malnutrition, which can lead to osteopenia and sarcopenia [21]. Investigators have reported that the incidence of osteosarcopenia was 25.8%, 28.3%, 27.5%, and 11.9% in patients with colorectal, pancreatic, extrahepatic cholangiocarcinoma, and hepatocellular carcinoma, respectively [7, 13, 22, 23]. Before treatment, 38% of the patients with esophageal cancer included in this study had osteosarcopenia, indicating that patients with esophageal cancer have a higher incidence of osteosarcopenia than patients with other carcinomas. Because patients with malignant tumors of the upper gastrointestinal tract are prone to impaired transit and absorption from an early stage [24], the incidence of osteosarcopenia may also be higher.

Many reports suggest that patients who respond histopathologically to CRT have a better prognosis than non-responders [25]. In the JCOG 0909 trial, the results of radical CRT for locally advanced esophageal cancer were comparable to those of standard therapy [26]. If CRT can be used to predict response to treatment, surgery may be employed as a salvage therapy rather than a planned treatment.

However, in this study, no significant differences in OS and RFS were observed on the basis of the presence or absence of osteosarcopenia. Moreover, osteosarcopenia was not identified as a prognostic factor in the univariate or multivariate analyses of OS and RFS. The JCOG1109 trial compared preoperative three-drug chemotherapy with a regimen of preoperative two-drug chemotherapy combined with radiotherapy, showing better OS in the DCF group, where only 20% achieved Grade 3 responses, than in the CF-RT group, where approximately half achieved Grade 3 responses [27]. This result indicates that the efficacy of NAC-RT does not necessarily directly reflect prognosis. While the presence of osteosarcopenia was not a prognostic factor in surgeries following NAC-RT, it could potentially serve as a prognostic factor for long-term outcomes after definitive CRT without surgery by influencing pathological response rates.

Inflammation stimulates angiogenesis, which may affect immune surveillance and therapeutic response [28]. Consequently, various blood biomarkers have been reported as predictors of treatment response to CRT [29, 30]. In this study, we also validated the PNI, NLR, PLR, LMR, CAR, PIV, and SII blood biomarkers. However, none of the blood biomarkers exhibited a correlation with histopathologic treatment response or were predictors of treatment in univariate or multivariate analyses. A possible explanation for this is the small sample size.

BMI has been reported as a prognostic factor for radical chemoradiotherapy in esophageal cancer [31]. Tumor depth and SCC antigen levels have also been reported to be predictive of response to preoperative chemotherapy and chemoradiation in esophageal cancer [32, 33]. The present study, consistent with previous research, revealed that tumor depth and SCC antigen correlated with the histopathologic response to NACRT. Additionally, BMI and osteosarcopenia correlated with the histopathological response to NACRT. Although osteosarcopenia was identified as an independent factor in the histopathological response to NACRT, previous studies have reported that factors related to tumor grade, such as tumor depth, differentiation, lymph node metastasis, and tumor size, are correlated with treatment response [34, 35]. There are reports that cancer metastasis is more likely to occur in a bone marrow microenvironment with reduced bone density [36], and that sarcopenia is closely related to its contribution by tumorigenesis and cancer cell growth [37], indicating that osteosarcopenia is related to tumor grade. Shachar et al. reported that sarcopenia was associated with reduced chemotherapy responsiveness in patients with breast cancer [38], while Wendrich et al. demonstrated that sarcopenia contributed to dose-limiting toxicity in chemoradiotherapy for patients with head and neck cancer [39]. Additionally, Nakao et al. reported that osteoporosis served as a predictor of chemotherapy-induced neutropenia in colorectal cancer [40]. In this study, although osteosarcopenia was associated with reduced CRT responsiveness, no relationship was observed between osteosarcopenia and dose reduction, discontinuation of CRT, or neutropenia.

Predicting the efficacy of NACRT in clinical practice involves numerous factors, including marker selection, evaluation methods, biopsy methodology, and timing of testing. It is challenging to develop an optimal prediction system without addressing these critical issues; however, the prediction of efficacy using imaging studies could address these concerns. Therefore, osteosarcopenia may serve as a simple and promising marker for predicting treatment response to CRT in esophageal cancer.

Nevertheless, the study has some limitations. The retrospective, single-center design of this study did not eliminate selection bias. In addition, the short observation period of 41 months may have been insufficient to adequately assess the effect of chemoradiotherapy and osteosarcopenia. Therefore, larger, longer-term prospective studies are needed to validate the findings of this study.

## Conclusion

Osteosarcopenia is a potential predictor of the pathological response to NACRT in esophageal cancer.

## Data Availability

No datasets were generated or analysed during the current study.
